# Supplementary material for: Development of an Image Analysis-Based Prognosis Score Using Google’s Teachable Machine in Melanoma
Source: Cancers (Basel). 2022 Apr 29;14(9):2243. doi: 10.3390/cancers14092243 (PMC9105888; doi:10.3390/cancers14092243)
Supplement: Supplementary file 1 [file cancers-14-02243-s001.zip › cancers-1690848-supplementary.pdf]

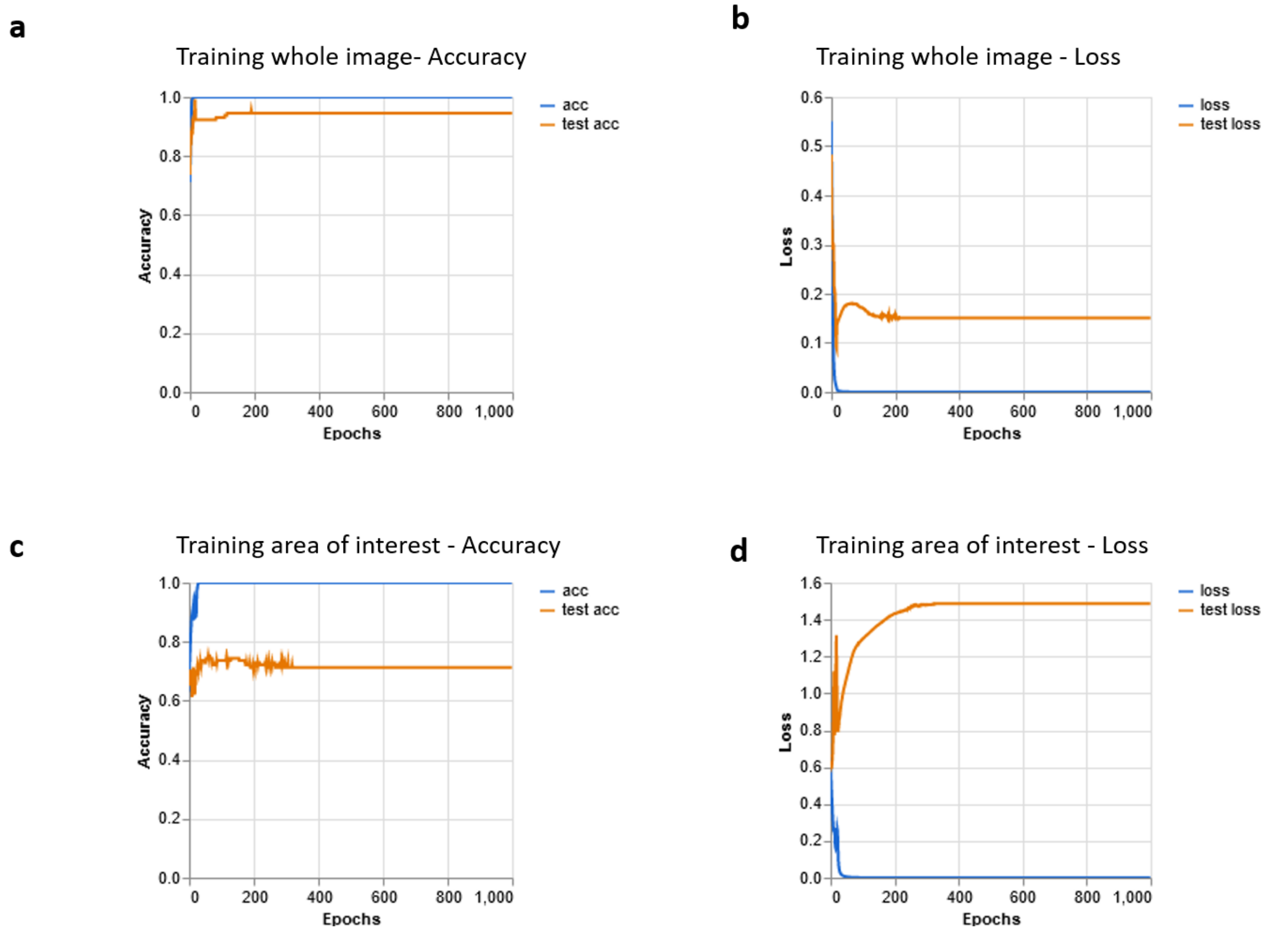


**Figure S1.** Training curves for accuracy (a+c) and loss (b+d). (**a**,**b**): Training curves for analysis of whole images. (**c**,**d**): Training curves for analysis of area of interest.

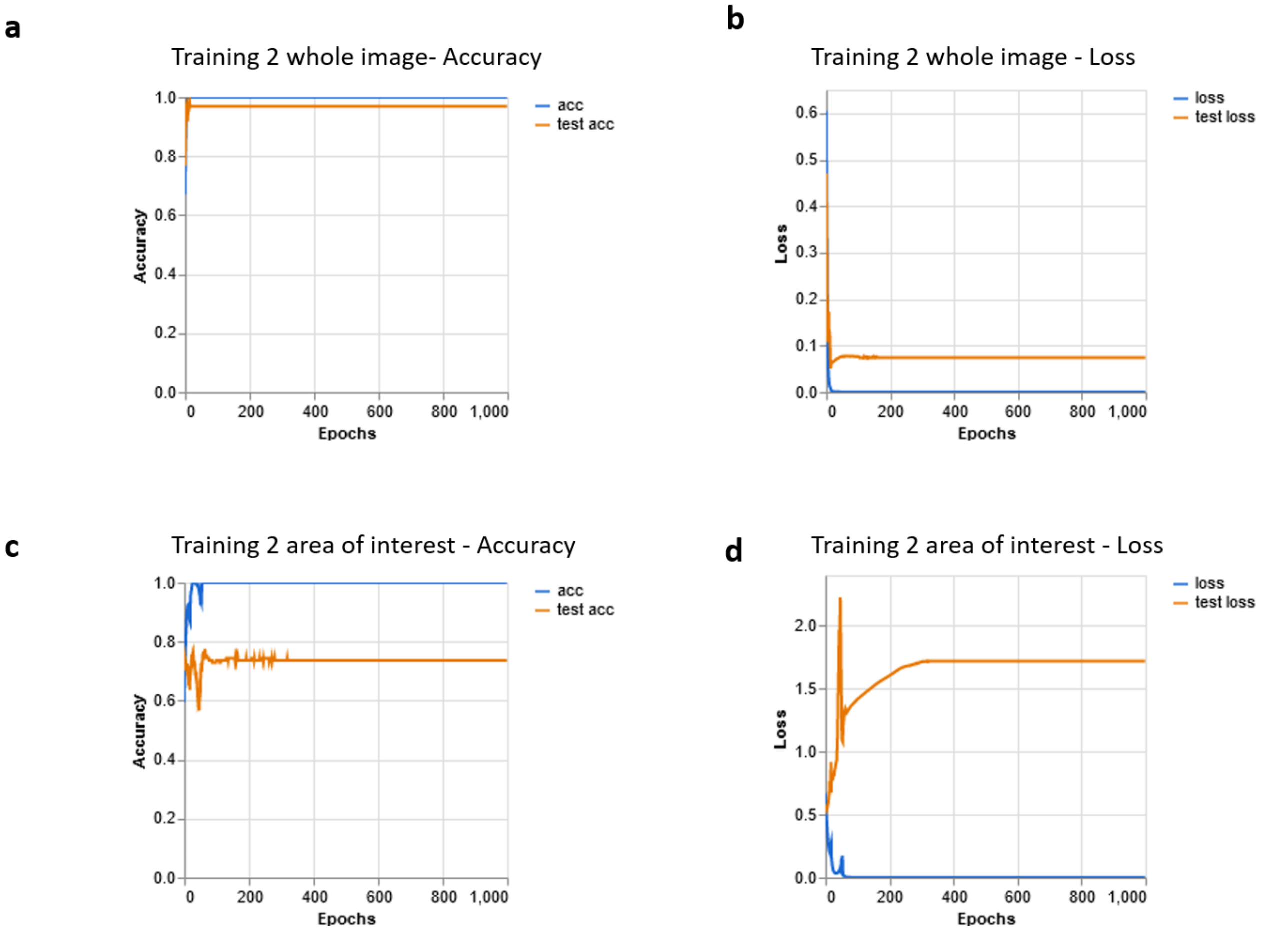


**Figure S2.** Training curves of repeated training for accuracy (a+c) and loss (b+d). (**a**,**b**): Training curves for analysis of whole images. (**c**,**d**): Training curves for analysis of area of interest.
